# CRL4^DCAF1^ ubiquitin ligase regulates PLK4 protein levels to prevent premature centriole duplication

**DOI:** 10.26508/lsa.202402668

**Published:** 2024-03-15

**Authors:** Josina Grossmann, Anne-Sophie Kratz, Alina Kordonsky, Gali Prag, Ingrid Hoffmann

**Affiliations:** 1 Cell Cycle Control and Carcinogenesis, German Cancer Research Center, DKFZ, Heidelberg, Germany; 2 Faculty of Biosciences, Heidelberg University, Heidelberg, Germany; 3 School of Neurobiology, Biochemistry and Biophysics, The George S. Wise Faculty of Life Sciences, Tel Aviv University, Tel Aviv, Israel; 4 Sagol School of Neuroscience, Tel Aviv University, Tel Aviv, Israel

## Abstract

Centrioles play important roles in the assembly of centrosomes and cilia. Centriole duplication occurs once per cell cycle and is dependent on polo-like kinase 4 (PLK4). To prevent centriole amplification, which is a hallmark of cancer, PLK4 protein levels need to be tightly regulated. Here, we show that the Cullin4A/B-DDB1-DCAF1, CRL4^DCAF1^, E3 ligase targets PLK4 for degradation in human cells. DCAF1 binds and ubiquitylates PLK4 in the G2 phase to prevent premature centriole duplication in mitosis. In contrast to the regulation of PLK4 by SCF^β-TrCP^, the interaction between PLK4 and DCAF1 is independent of PLK4 kinase activity and mediated by polo-boxes 1 and 2 of PLK4, suggesting that DCAF1 promotes PLK4 ubiquitylation independently of β-TrCP. Thus, the SCF^Slimb/β-TrCP^ pathway, targeting PLK4 for ubiquitylation based on its phosphorylation state and CRL4^DCAF1^, which ubiquitylates PLK4 by binding to the conserved PB1-PB2 domain, appear to be complementary ways to control PLK4 abundance to prevent centriole overduplication.

## Abstract (precis)

This study identifies the E3 ubiquitin ligase, CRL4DCAF1, as a new regulator of polo-like kinase 4, PLK4, protein levels in the G2 phase of the cell cycle to prevent premature duplication of centrioles.

## Introduction

Centriole biogenesis has to be tightly controlled to prevent aberrant centrosome number, which can lead to chromosome missegregation and aneuploidy and has been associated with cancer (Nigg & Holland, 2018). Centriole duplication is triggered by and dependent on polo-like kinase 4 (PLK4), a divergent member of the PLK family (Bettencourt-Dias et al, 2005; Habedanck et al, 2005). Human PLK4 is localized to centrosomes through interaction with two receptors, CEP152 and CEP192 (Cizmecioglu et al, 2010; Dzhindzhev et al, 2010; Hatch et al, 2010; Kim et al, 2013; Sonnen et al, 2013). Binding of PLK4 to its centriole substrate STIL promotes activation of the kinase (Ohta et al, 2014; Lopes et al, 2015; Moyer et al, 2015). PLK4 phosphorylates STIL in a conserved STAN motif, which leads to binding and recruitment of SAS6 (Dzhindzhev et al, 2010; Ohta et al, 2014; Kratz et al, 2015; Moyer et al, 2015), which is necessary for cartwheel assembly (Nakazawa et al, 2007).

PLK4 protein levels are regulated by ubiquitylation and proteasomal degradation. Previous work has revealed that this is in part mediated by the SCF (Skp1/Cullin/F-box) β-TrCP/Slimb E3 ubiquitin ligase (Cunha-Ferreira et al, 2009; Rogers et al, 2009; Guderian et al, 2010). The β-propeller of the F-box protein β-TrCP recognizes a conserved phosphodegron in the N-terminal PEST motif of PLK4, which is generated by homodimer-dependent trans-autophosphorylation of human PLK4 (Guderian et al, 2010; Holland et al, 2010; Cunha-Ferreira et al, 2013; Klebba et al, 2013). The E3 ubiquitin ligase MIB1 is also implicated in regulating PLK4 levels, particularly under conditions of aberrant PLK4 expression (Cajanek et al, 2015).

CUL4-RING ligases contain the scaffold proteins CUL4A or CUL4B, which are conserved from yeast to humans. They bind to a substrate-targeting unit, which is composed of the adaptor DNA damage–binding protein 1 (DDB1) and a member of the DDB1- and CUL4-associated factors (DCAFs), a family of WD40 repeat proteins that confer substrate specificity (Jackson & Xiong, 2009). Among the DCAFs, DCAF1 is a critical substrate receptor in the CUL4-DDB1-DCAF1 complex (Han et al, 2020). DCAF1 is also known as Vpr-binding protein (VprBP), as it was initially discovered as a target protein hijacked by the viral protein Vpr of HIV-1 (Tan et al, 2007). DCAF1 is involved in a number of fundamental cellular processes including DNA replication (McCall et al, 2008) and cell cycle regulation (Guo et al, 2016). A number of substrates of the CUL4-DDB1-DCAF1 complex CRL4^DCAF1^ have been described. Among those are p53 (Hrecka et al, 2007; Guo et al, 2016), the replication factor MCM10 (Kaur et al, 2012), and protein phosphatase 2A (Yu et al, 2015). The activity of the CUL4-DDB1-DCAF1 complex itself is regulated by its oligomerization state (Mohamed et al, 2021).

Recent data showed that a β-TrCP–binding mutant of PLK4 was still ubiquitylated and only modestly stabilized in human cells, suggesting that additional ubiquitin ligases might regulate PLK4 protein levels in canonical centriole duplication (Rogers et al, 2009; Holland et al, 2010; Klebba et al, 2013). Here, we report that the CRL4^DCAF1^ E3 ubiquitin ligase complex contributes to the regulation of PLK4 abundance. DCAF1 binds to and promotes ubiquitylation of PLK4. AlphaFold2.0 modeling corroborated by in vivo analysis demonstrates a novel binding interface in which the unstructured DCAF1 acidic tail binds to the conserved positive grooves of the PLK4 polo-boxes 1 and 2. Furthermore, we find that the CRL4^DCAF1^ complex controls PLK4 levels in the G2 phase, when β-TrCP activity is low (Paul et al, 2022), thus preventing premature centriole disengagement and centriole duplication. The interaction between DCAF1 and PLK4 and the ubiquitylation of PLK4 occur in a PLK4 kinase activity–independent and phosphorylation-independent manner, suggesting that the SCF^β-TrCP^ and the CRL4^DCAF1^ E3 ubiquitin ligases independently control PLK4 protein abundance and therefore centriole duplication.

## Results

### CUL4-DDB1-DCAF1 E3 ubiquitin ligase, CRL4^DCAF1^, regulates PLK4 protein levels

**Figure S1.**
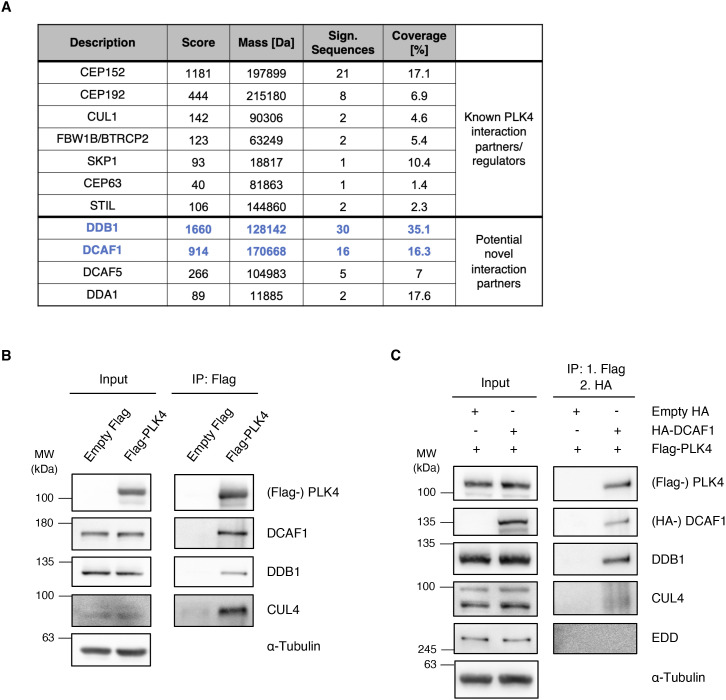
A screen for PLK4 interaction partners identifies DCAF1, a component of the CRL4 E3 ubiquitin ligase. **(A)** Mass spectrometry analysis of Flag-PLK4 IP samples identified known PLK4 interaction partners, substrates, or regulators, and potential novel interaction partners. MASCOT score, molecular weight (mass), identified protein sequence, and coverage of selected candidates are shown. **(B)** Flag-PLK4 was overexpressed in HEK293T cells for 48 h. Co-precipitated E3 ligase complex components DCAF1, DDB1, and CUL4 were detected by IP against the Flag tag and subsequent Western blot analysis. **(C)** Flag-PLK4 and HA-DCAF1 were overexpressed in HEK293T cells for 48 h. The presence of DBB1 and CUL4 in the same complex was analyzed by IP against the Flag tag, followed by Flag peptide elution, subsequent IP against the HA tag using the eluates, and Western blot analysis of the double IP samples.

**Figure 1.**
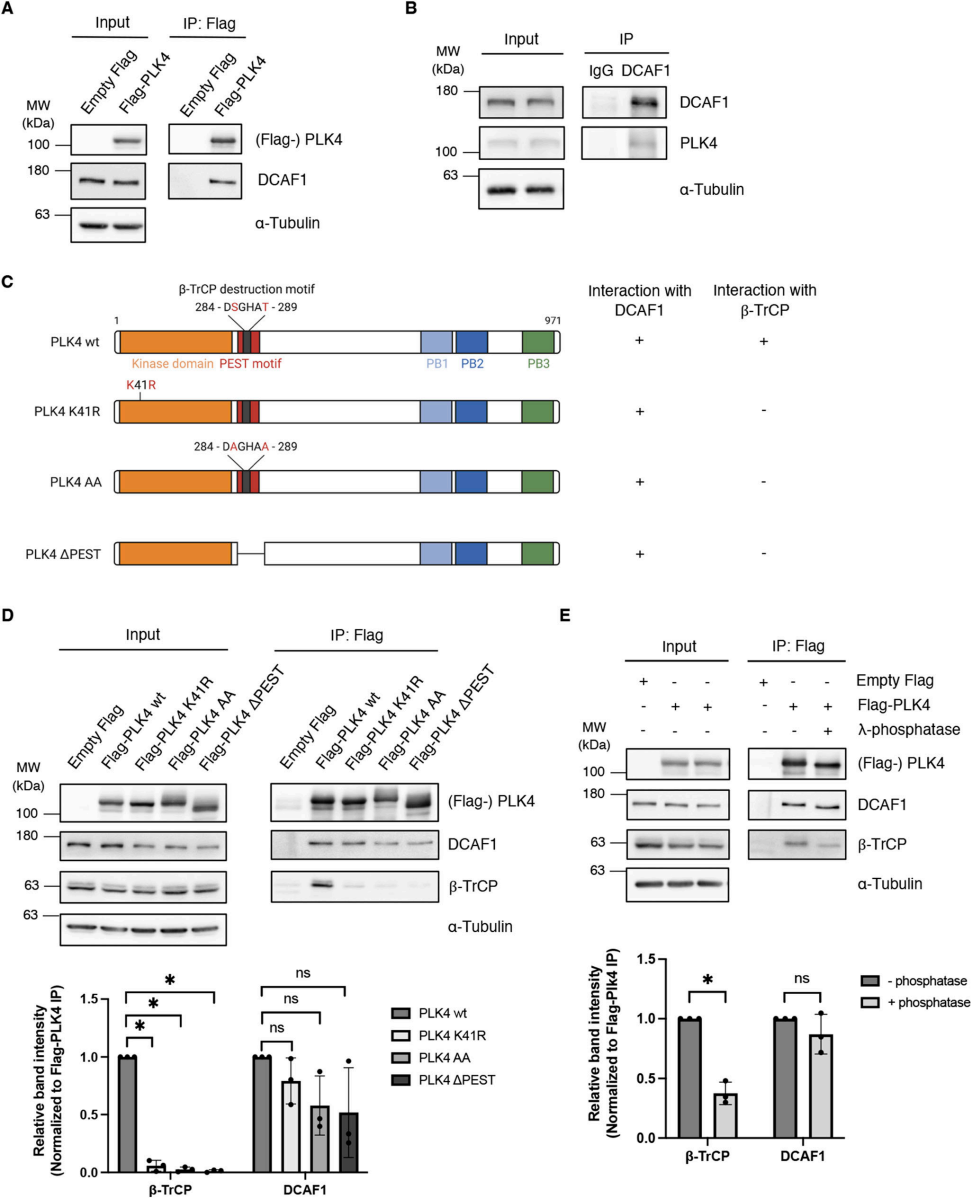
Polo-like kinase 4 (PLK4) interacts with DCAF1 independently of PLK4 kinase activity and phosphorylation. **(A)** Flag-PLK4 was overexpressed in HEK293T cells for 48 h. Co-precipitated DCAF1 was detected by IP against the Flag tag and subsequent Western blot analysis. **(B)** Endogenous DCAF1 was immunoprecipitated from HEK293T cell lysates using unspecific IgG control or specific DCAF1 antibodies and protein G Sepharose. **(C)** Overview of PLK4 mutants used in (D). Interaction of the PLK4 mutants with endogenous DCAF1 and β-TrCP is indicated on the right (− and +). **(D)** Flag-PLK4 wt or Flag-PLK4 mutants (K41R, AA, ΔPEST) were overexpressed in HEK293T cells for 48 h. Co-precipitated DCAF1 and β-TrCP were detected by IP against the Flag tag and subsequent Western blot analysis. Quantification of relative β-TrCP/Flag-PLK4 or DCAF1/Flag-PLK4 signal normalized to Flag-PLK4 wt, N = 3. *P < 0.05 and ns *P* > 0.05. Data are presented as the mean ± SD. **(E)** Flag-PLK4 was overexpressed in HEK293T cells for 48 h. Co-precipitated DCAF1 and β-TrCP were detected by IP against the Flag tag with or without dephosphorylation of protein samples using λ-phosphatase and subsequent Western blot analysis. Quantification of relative β-TrCP/Flag-PLK4 or DCAF1/Flag-PLK4 signal normalized to control, N = 3. *P < 0.05 and ns *P* > 0.05. Data are presented as the mean ± SD.

**Figure S2.**
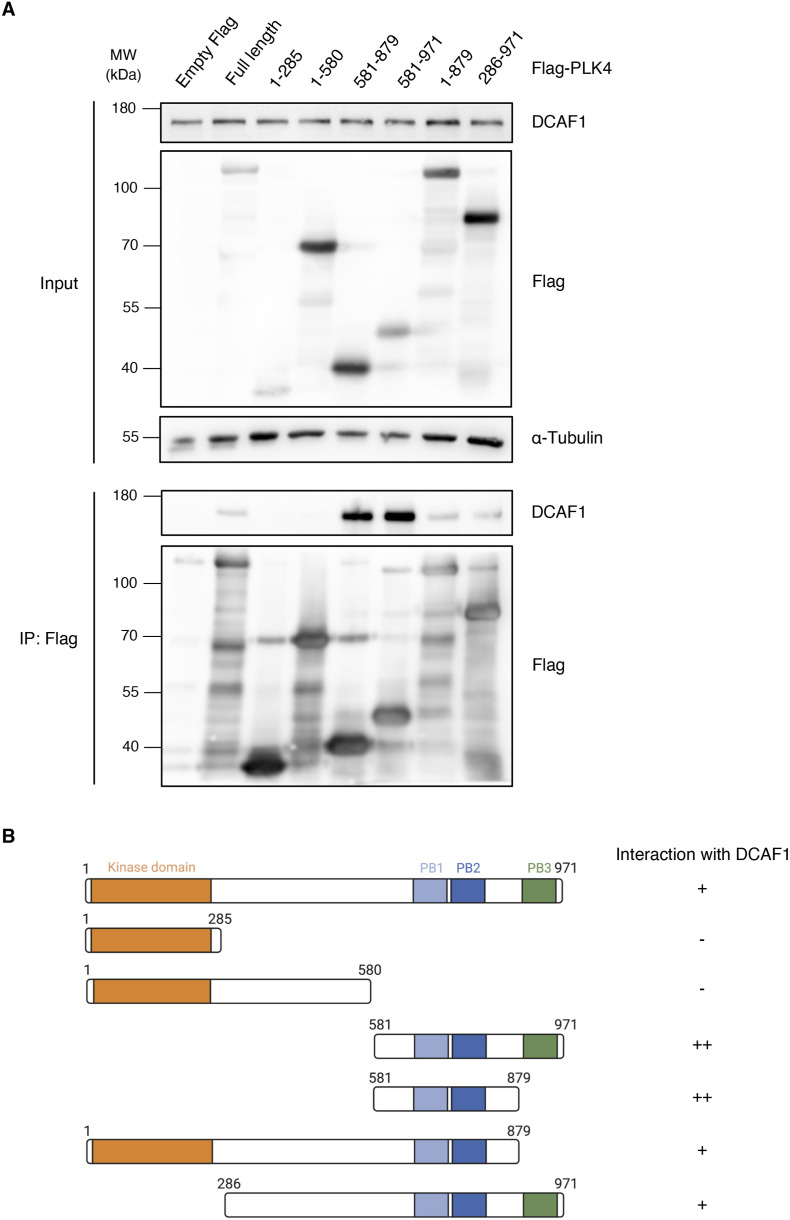
DCAF1 binds to PB1-2 of PLK4. **(A)** Flag-PLK4 full-length or different truncated fragments were overexpressed in HEK293T cells for 48 h. Co-precipitated DCAF1 was detected by IP against the Flag tag and subsequent Western blot analysis. **(B)** Overview of the different Flag-PLK4 fragments used in (A). Interaction of the fragments with endogenous DCAF1 is indicated on the right (−, +, and ++).

To identify ubiquitin ligases that regulate PLK4 protein levels, we performed co-immunoprecipitation experiments with PLK4 as bait followed by subsequent mass spectrometry analysis (Fig S1A). Interestingly, among other known PLK4-interacting proteins such as STIL, CEP152, and β-TrCP, we identified the substrate recognition component DCAF1 (VprBP), along with DDB1, a core component of CUL4A- and CUL4B-based E3 ubiquitin ligases (Jackson & Xiong, 2009). We confirmed the interaction between overexpressed PLK4 and endogenous DCAF1 (Fig 1A). In addition, an interaction between endogenous PLK4 and DCAF1 was observed using specific antibodies (Fig 1B). Apart from its interaction with DCAF1, we found that PLK4 also interacted with DDB1 and CUL4 (Fig S1B). Sequential co-immunoprecipitation experiments revealed that PLK4 exists in a complex with DCAF1, DDB1, and CUL4 but not with UBR5/EDD, a HECT-type E3 ubiquitin ligase that also contains a β-propeller as a substrate-binding domain (Fig S1C) (Maddika & Chen, 2009). DCAF1 is a centrosome protein (Hossain et al, 2017), and DDB1 was previously identified in a proteomic approach to define the constituents of human centrosomes (Andersen et al, 2003). Autophosphorylation of two amino acid residues (S285/T289 in human PLK4) within the PLK4 β-TrCP–binding motif promotes the binding of β-TrCP and subsequent ubiquitylation and destruction of PLK4 (Guderian et al, 2010; Holland et al, 2010; Cunha-Ferreira et al, 2013; Klebba et al, 2013). As β-TrCP and DCAF1 share a similar β-propeller substrate receptor domain, we first aimed at determining whether DCAF1 would bind to the same or a different site within PLK4 than β-TrCP and whether this binding is dependent on the autophosphorylation of PLK4. To address this question, we used several mutants of PLK4. First, we generated a kinase-dead version of PLK4 by a mutation within the kinase domain (K41R) (Bahtz et al, 2012); second, we mutated either the β-TrCP recognition motif in PLK4 to AA (S285A + T289A); or third, we deleted the PEST destruction motif (Fig 1C). Whereas binding between PLK4 and β-TrCP is lost when PLK4 is kinase-dead or mutated in the recognition motif, DCAF1 interacted with the kinase-dead PLK4 mutant, the β-TrCP–binding mutant PLK4-AA, and the PLK4-ΔPEST mutant to a similar level as with WT PLK4 (Fig 1D). Consistent with this, using the indicated PLK4 fragments (Fig S2B), we could clearly map the binding site of DCAF1 to a C-terminal fragment of PLK4 containing the tandem polo-boxes (PB1-PB2) (Fig S2A). To further prove that binding between PLK4 and DCAF1 is independent of PLK4 autophosphorylation or phosphorylation by any other unknown kinase, we treated immunoprecipitated PLK4 with λ-phosphatase and found that although binding to β-TrCP was reduced, no reduction in DCAF1 binding to PLK4 could be observed (Fig 1E). Together, these data suggest that autophosphorylation of PLK4 or phosphorylation of PLK4 in general is not required for the interaction between DCAF1 and PLK4.

**Figure 2.**
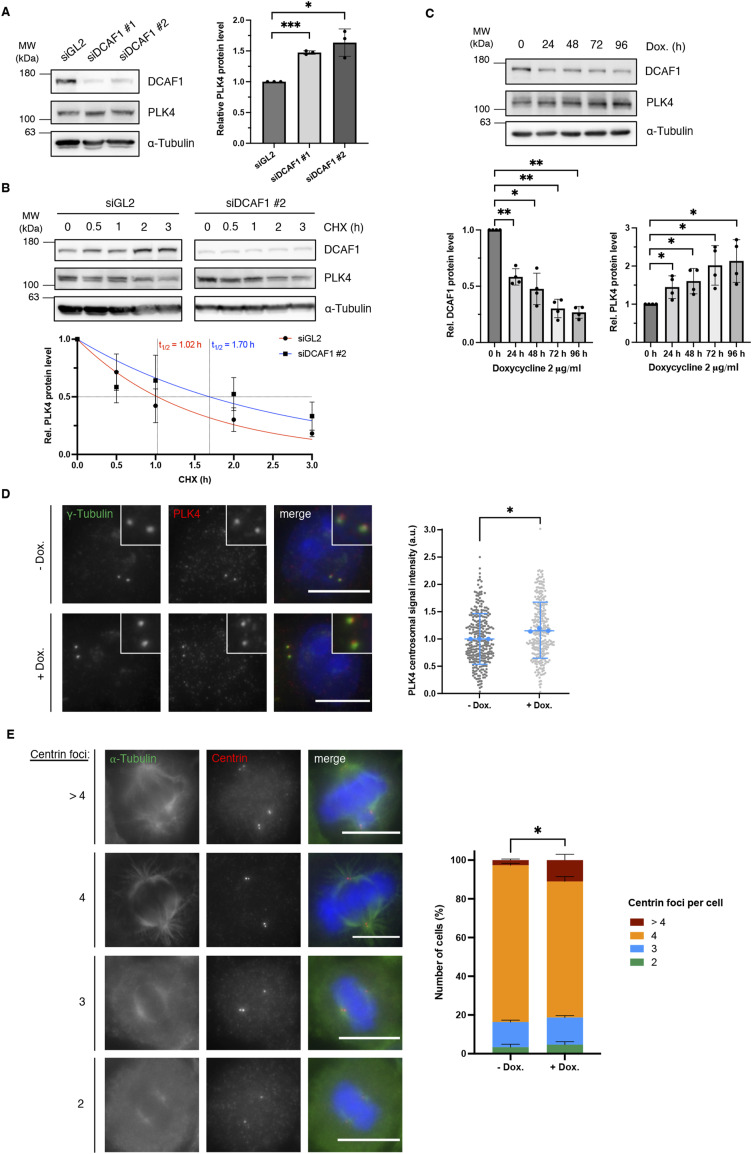
DCAF1 knockdown increases polo-like kinase 4 (PLK4) protein levels and promotes the formation of supernumerary centrioles in mitosis. **(A)** U2OS cells were transfected twice with siRNA against either GL2 (control) or DCAF1 and harvested 72 h after the first transfection. Protein levels were determined by Western blot analysis. Quantification of relative PLK4/α-tubulin signal normalized to siGL2, N = 3. ***P < 0.001 and *P < 0.05. Data are presented as the mean ± SD. **(B)** U2OS cells were transfected twice with siRNA against either GL2 (control) or DCAF1, and protein synthesis was blocked 72 h after the first transfection by treatment with 100 μg/ml cycloheximide for the indicated durations before harvest. Protein half-lives were determined by nonlinear fit to a one-phase decay model. N = 3. **(C)** HeLa tet-on shDCAF1 cells were treated with 2 μg/ml doxycycline for the indicated durations before harvest. Protein levels were determined by Western blot analysis. Quantification of relative DCAF1/α-tubulin and PLK4/α-tubulin signal normalized to 0-h time point, N = 4. **P < 0.01 and *P < 0.05. Data are presented as the mean ± SD. **(D)** For knockdown of DCAF1, HeLa tet-on shDCAF1 cells were treated with 2 μg/ml doxycycline for 72 h before fixation. For immunofluorescence analysis, cells were stained with antibodies against γ-tubulin and PLK4. Scale bar: 10 μm. Centrosomal signal intensities were quantified, and background fluorescence intensity was subtracted. Values were normalized to the untreated control. Individual values are presented with the mean ± SD. In total, n = 300 centrosomes per condition were analyzed in N = 3 independent experiments. Statistical analysis of the mean values of three experiments. *P < 0.05. **(E)** For knockdown of DCAF1, HeLa tet-on shDCAF1 cells were treated with 2 μg/ml doxycycline for 72 h before fixation. For immunofluorescence analysis, cells were stained with antibodies against α-tubulin and centrin. Scale bar: 10 μm. The number of centrioles per mitotic cell was determined based on centrin staining. N = 3 independent experiments with 100 mitotic cells per condition in each experiment. *P < 0.05 for >4 centrin foci. Data are presented as the mean + SD.

**Figure S3.**
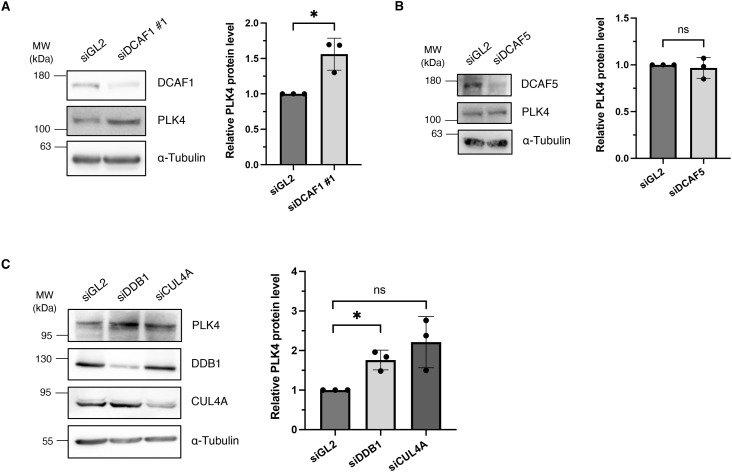
Depletion of DCAF1, DDB1 and CUL4A leads to an increase in PLK4 protein levels. **(A)** HEK293T cells were transfected twice with siRNA against either GL2 (control) or DCAF1 and harvested for 72 h after the first transfection. Protein levels were determined by Western blot analysis. Quantification of relative polo-like kinase 4 (PLK4)/α-tubulin signal normalized to siGL2, N = 3. *P < 0.05. Data are presented as the mean ± SD. **(B)** HEK293T cells were transfected twice with siRNA against either GL2 (control) or DCAF5 and harvested 72 h after the first transfection. Protein levels were determined by Western blot analysis. Quantification of relative PLK4/α-tubulin signal normalized to siGL2, N = 3. ns *P* > 0.05. Data are presented as the mean ± SD. **(C)** HeLa cells were transfected twice with siRNA against either GL2 (control), DDB1, or CUL4A and harvested 72 h after the first transfection. Protein levels were determined by Western blot analysis. Quantification of relative PLK4/α-tubulin signal normalized to siGL2, n = 3. *P < 0.05 and ns *P* > 0.05. Data are presented as the mean ± SD.

**Figure S4.**
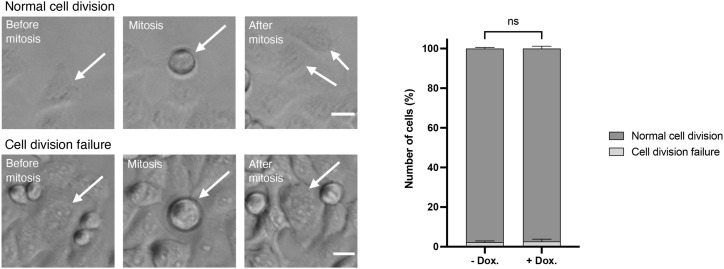
Images from live-cell imaging performed with HeLa tet-on shDCAF1 cells showing a normal cell division and a cell division failure. White arrows indicate representative cells. Scale bar: 20 μm. N = 3 independent experiments with n = 100 mitoses analyzed per condition for each experiment. ns *P* > 0.05. Data are presented as the mean + SD.

We further investigated whether DCAF1 depletion would affect PLK4 protein turnover. Treatment of cells with DCAF1-specific siRNAs led to an increase in PLK4 protein levels (Figs 2A and S3A). The increase in PLK4 protein levels was not detectable upon depletion of another DCAF family member, DCAF5 (Zhang et al, 2019), which was also identified in our screen (Figs S1A and S3B). Depletion of other CRL4 complex components, such as the adaptor protein DDB1, also led to a significant increase in PLK4 protein levels (Fig S3C). Treatment of DCAF1-depleted cells with cycloheximide (CHX) to block protein translation led to stabilization of PLK4 and a slight increase in protein half-life (Fig 2B). The effect on PLK4 protein levels was also clearly visible when we depleted DCAF1 in a doxycycline-inducible HeLa cell line. The time-dependent decrease in DCAF1 protein levels resulted in an increase in PLK4 protein levels (Fig 2C). Down-regulation of DCAF1 also led to an increase in PLK4 protein levels at the centrosome (Fig 2D). We anticipate that increased PLK4 protein levels upon depletion of DCAF1 should trigger centriole overduplication (Habedanck et al, 2005). To assess this hypothesis, we depleted DCAF1 by adding doxycycline and observed supernumerary centrioles leading to the formation of multipolar spindles in mitosis (Fig 2E). PLK4 has been implicated in the regulation of cytokinesis (Rosario et al, 2010; Press et al, 2019), and cytokinesis failure might be another cause for the formation of multiple centrioles. To exclude the possibility that the observed effect is due to cytokinesis failure, we verified by live-cell imaging that cell division was not impaired in the absence of DCAF1 (Fig S4). Our data therefore suggest that CRL4^DCAF1^ might function to keep PLK4 protein levels low, thus preventing centriole overduplication.

### DCAF1 interacts with and ubiquitylates PLK4

**Figure S5.**
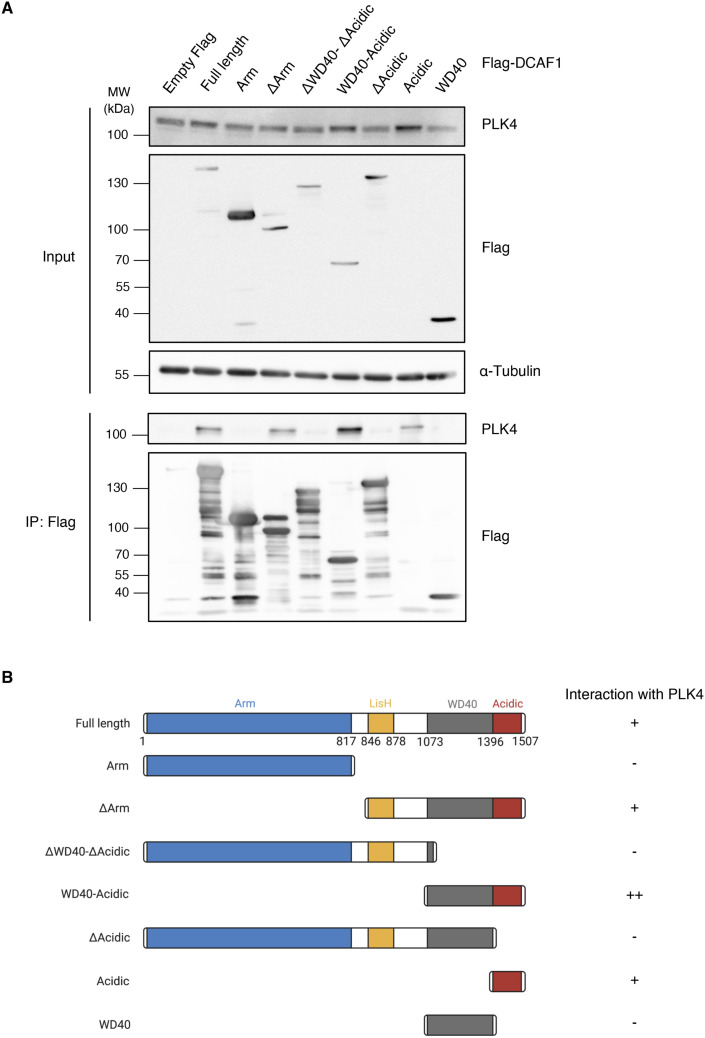
PLK4 binds to the C-terminal acidic domain of DCAF1. **(A)** Flag-DCAF1 full-length or different truncated fragments were overexpressed in HEK293T cells for 48 h. Co-precipitated polo-like kinase 4 was detected by IP against the Flag tag and subsequent Western blot analysis. **(B)** Overview of the different Flag-DCAF1 fragments used in (A). Interaction of the fragments with endogenous polo-like kinase 4 is indicated on the right (−, +, and ++).

To identify the minimal domain of DCAF1 that binds to PLK4, we used truncated, Flag-tagged fragments of DCAF1 (Cassiday et al, 2015) (Fig S5B) and performed co-immunoprecipitation experiments. Interestingly, we found that the unstructured acidic domain (Acidic) of DCAF1, located downstream of the WD40/β-propeller domain at the C-terminal end, mediates the binding to PLK4 (Fig S5A and B). In addition, PLK4 strongly binds to a WD40-Acidic motif (aa 1073-1507) but not to the WD40 domain of DCAF1 alone (aa 1073-1396), suggesting that both domains together might contribute to PLK4 binding. It is conceivable that the acidic domain is the major interactor, whereas the contribution of the WD40 domain is only minor by most likely having a stabilizing effect on the acidic domain.

**Figure S6.**
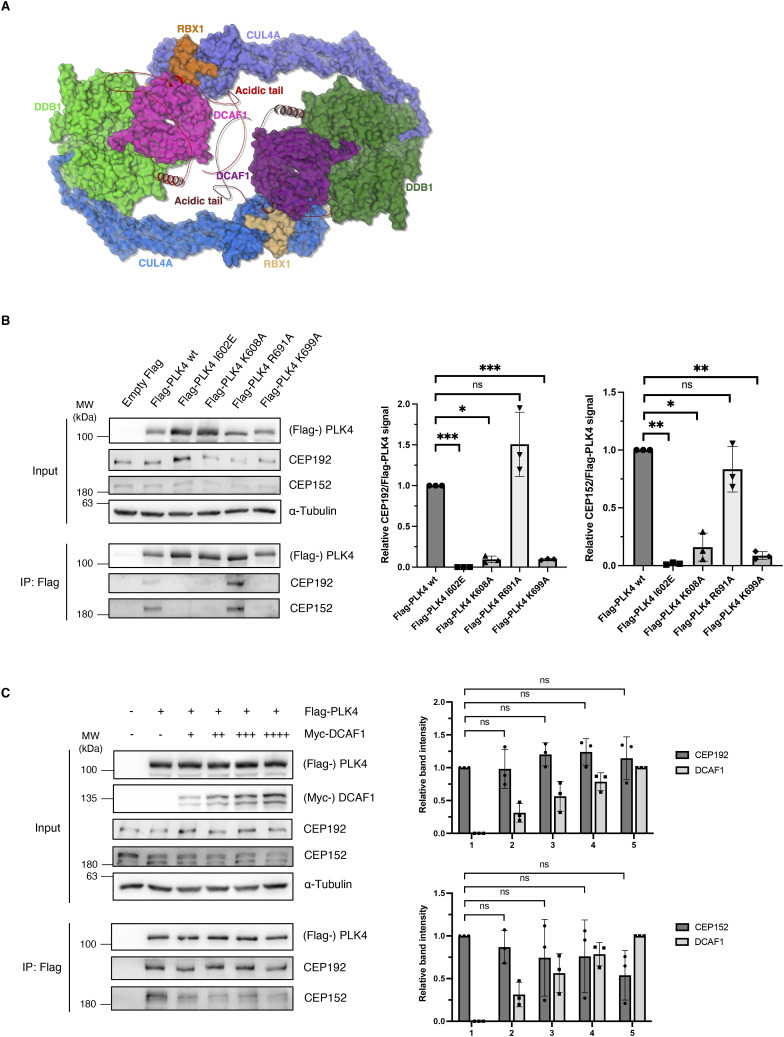
DCAF1, CEP152 and CEP192 can bind simultaneously to the PB1-2 of PLK4. **(A)** Cryo-EM structure of the CRL4^DCAF1^ complex and the AlphaFold model of the DCAF1 acidic domain. The dimeric structure of DCAF1 (7OKQ) is shown, and the names of protein components are indicated with the same color code. The unstructured acidic C-terminal tails of DCAF1 are shown in red and ruby. **(B)** Indicated Flag-PLK4 mutants were overexpressed in HEK293T cells for 48 h. Co-precipitated CEP192 and CEP152 were detected by IP against the Flag tag and subsequent Western blot analysis. Quantification of relative CEP192/Flag-PLK4 and CEP152/Flag-PLK4 signal normalized to Flag-PLK4 wt, N = 3. ***P < 0.001, **P < 0.01, *P < 0.05, and ns *P* > 0.05. Data are presented as the mean ± SD. **(C)** Flag-PLK4 was overexpressed in HEK293T cells together with increasing amounts of Myc-DCAF1 plasmid DNA in different samples (+, ++, +++, ++++) for 48 h. Co-precipitated CEP192 and CEP152 were detected by IP against the Flag tag and subsequent Western blot analysis. Quantification of relative CEP192/Flag-PLK4, CEP152/Flag-PLK4, and Myc-DCAF1 signal, N = 3 independent experiments. ns *P* > 0.05. Data are presented as the mean ± SD.

**Figure 3.**
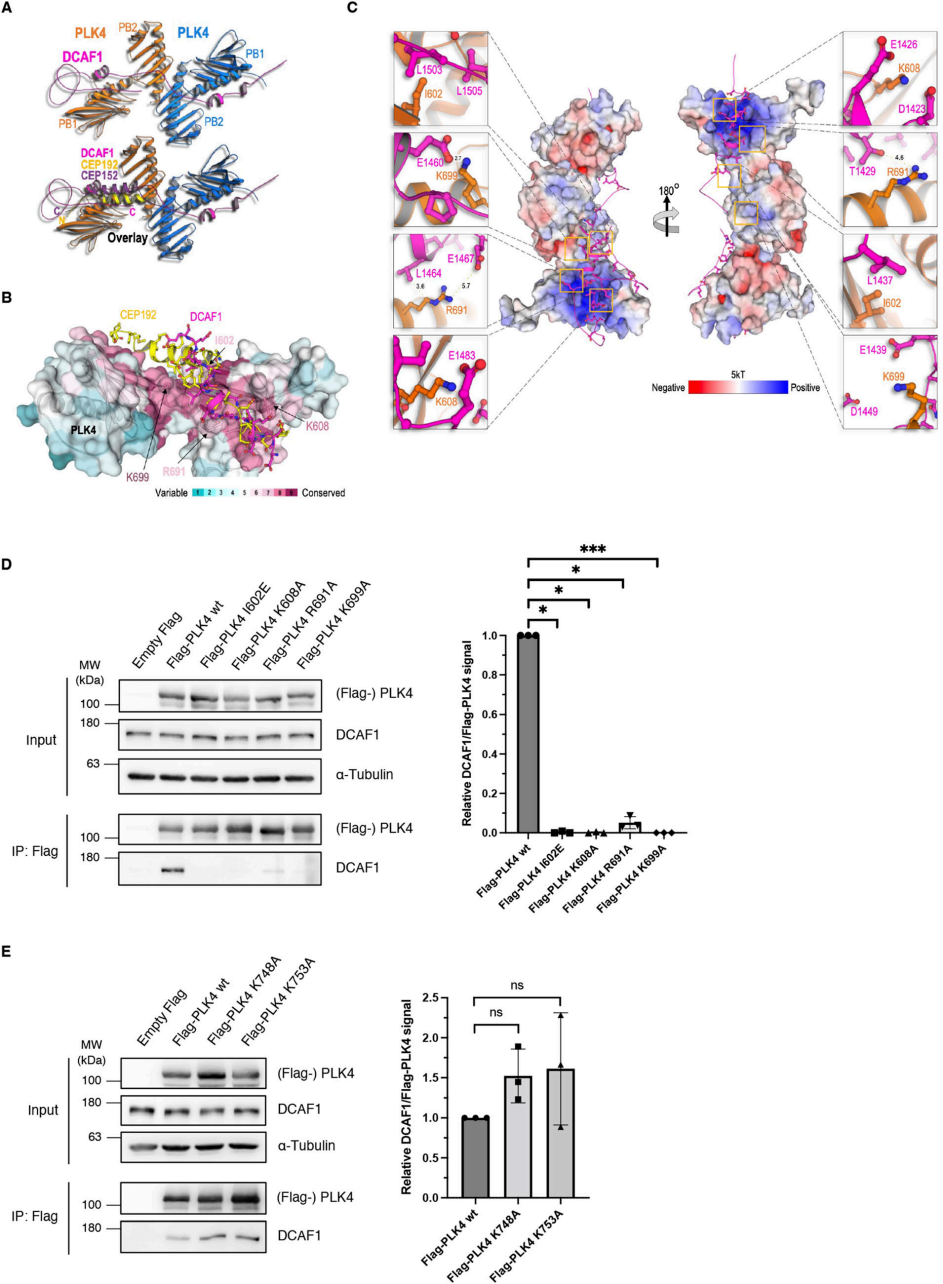
Structural model of the DCAF1/PLK4 complex. **(A)** Model of the DCAF1/PLK4 complex was constructed by AlphaFold2.0. The model was further minimized by Refmac5 (idealization procedure) and examined by structural-based mutagenesis and binding assays. Structures of the CEP192/PLK4 complex (PDB 4N7Z) and the DCAF1/PLK4 complex, and the overlay of the two are shown. **(B)** DCAF1 and CEP192 bind a conserved groove in PLK4. Conservation analysis was used to render the conservation level of the residues in the PB1/2 domain of PLK4, which is shown as a transparent surface to allow the view of residues that were mutated. CEP192 (yellow) and DCAF1 (magenta) are shown as cartoons with ball-and-stick residues. **(C)** Surface electrostatic potential was calculated by the Adaptive Poisson–Boltzmann Solver with the indicated kT. The acidic domain of DCAF1 (magenta cartoon) binds the positive (basic) groove of the PLK4 PB1/2 homodimers. **(D)** Indicated Flag-PLK4 mutants were overexpressed in HEK293T cells for 48 h. Co-precipitated DCAF1 was detected by IP against the Flag tag and subsequent Western blot analysis. Quantification of relative DCAF1/Flag-PLK4 signal normalized to Flag-PLK4 wt, N = 3. ***P < 0.001 and *P < 0.05. Data are presented as the mean ± SD. **(E)** Indicated Flag-PLK4 mutants were overexpressed in HEK293T cells for 48 h. Co-precipitated DCAF1 was detected by IP against the Flag tag and subsequent Western blot analysis. Quantification of relative DCAF1/Flag-PLK4 signal normalized to Flag-PLK4 wt, N = 3. ns *P* > 0.05. Data are presented as the mean ± SD.

**Figure S7.**
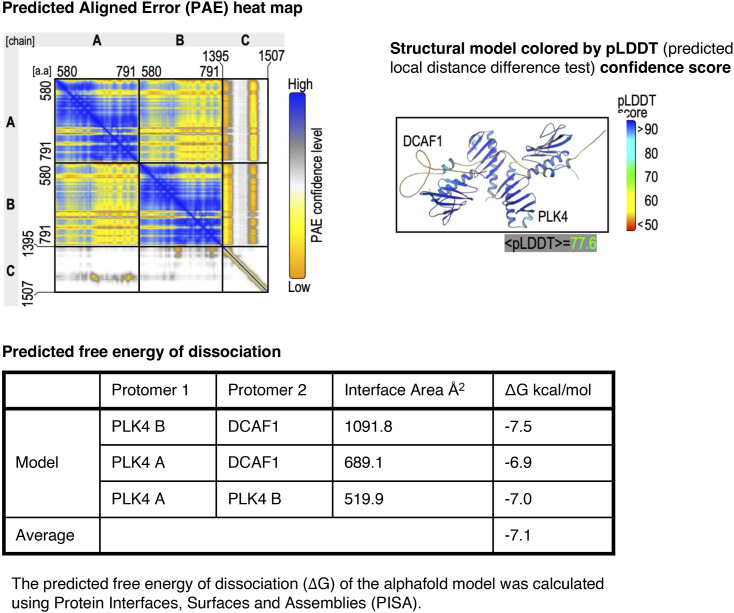
Internal model quality evaluation by AlphaFold. The PAE heat map shows the predicted alignment error between pair residues in the model. The four-color scale provides a contrast between high- and low-confidence regions. The structural model is shown on the right. This model is colored based on the predicted local distance difference test (pLDDT), which predicts the confidence by AlphaFold. The predicted free energy of dissociation was calculated and is shown below.

Next, we aimed at investigating the structure of the DCAF1/PLK4 complex more closely and constructed a model of the complex using AlphaFold2.0 (Jumper et al, 2021). Recently, the structure of CRL4^DCAF1^ was determined by cryo-EM (Mohamed et al, 2021). Although the 1.5-MDa structure provides atomic resolution insight into the mechanism of ligase assembly and activation, the DCAF1 C-terminal acidic tail was not observed. Usually, cryo-EM cannot detect highly mobile unstructured regions, which is consistent with our AlphaFold2.0 model, suggesting that the acidic tail alone is intrinsically unstructured (Fig S6A). AlphaFold2.0 enabled us to construct a model of the DCAF1/PLK4 complex which we compared with the CEP192(CEP152)/PLK4 complex (Fig 3A). The model shown in Fig S7 presented the highest structural prediction confidence score and the maximum of conserved residues in PLK4 that participate in DCAF1 binding and showed the highest similarity to the complexes of PLK4 with CEP152 and CEP192. We calculated the predicted free energy of dissociation using PISA (Krissinel & Hendrik, 2007). An overlay between the structures revealed that CEP192(CEP152) and DCAF1 bind to the same groove within the PLK4 PB1-2 (Fig 3B). The model suggests that a dimer of PLK4 binds to a single chain of the unstructured DCAF1 acidic domain. Interestingly, binding induces the formation of helices in the unstructured acidic region: the first helix (D1420-E1436) of DCAF1 positioned in a basic groove formed between the PB1 and PB2 of one protomer and the second helix (D1458-E1466) bound to the same basic groove of the second protomer in the PLK4 dimer (Fig 3C). The model demonstrated that an extended unstructured region (E1467-E1507), downstream of the second helix, wriggles back onto the PB1 surface of the second protomer. A connector region between the two DCAF1 helices forms interactions with a groove mainly of the second protomer. Residues at the PB binding grooves are highly conserved, indicating their importance for protein binding (Fig 3B). The grooves present positive charge surfaces as visualized by the calculated Adaptive Poisson–Boltzmann Solver (Baker et al, 2001) (Fig 3C).

To experimentally assess the importance of the residues located in the binding grooves for the PLK4-DCAF1 interaction in vivo, PLK4 constructs harboring point mutations in the PB1-2 domain (amino acids are depicted in orange in Fig 3C) were generated and transfected into HEK293T cells followed by immunoprecipitation. In alignment with the structural model, we found that binding of DCAF1 to these PLK4 mutants but not to PLK4 WT was abolished, suggesting that these amino acids are critical for binding (Fig 3D). In contrast, PLK4 mutations that are located near but not directly within the predicted interaction interface did not diminish the interaction with DCAF1, further confirming the structural model (Fig 3E). Interestingly, similar but not identical amino acids within the PLK4 PB1-2 are critical for binding to CEP152 and CEP192 (Fig S6B). In line with this result, competition assays in HEK293T cells revealed that increasing amounts of DCAF1 cannot prevent CEP152 or CEP192 from binding to PLK4, indicating that all three proteins can bind to the PB1-2 domain of PLK4 simultaneously (Fig S6C).

**Figure 4.**
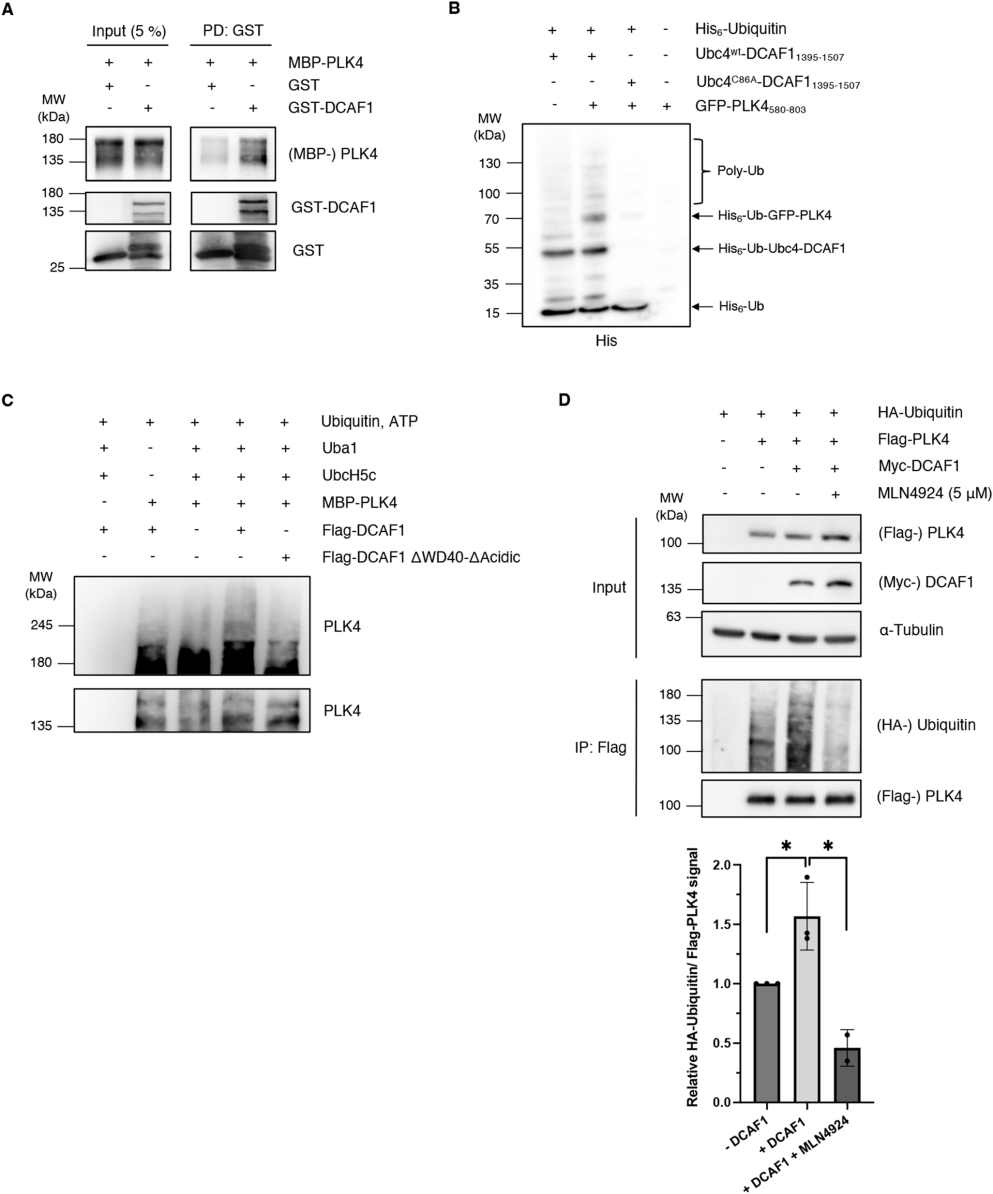
DCAF1 ubiquitylates polo-like kinase 4 (PLK4) in vitro and in vivo. **(A)** Purified recombinant GST-DCAF1 or empty GST was combined with MBP-PLK4, and GST pull-down assays were performed using glutathione CL-4B Sepharose beads. Input and eluate samples were analyzed by Western blot. **(B)** His-ubiquitin was co-expressed in *E. coli* together with a GFP-PLK4 construct containing the PB1-PB2 domain of PLK4 and a fusion construct consisting of the DCAF1 acidic domain and the E2 enzyme Ubc4, with or without mutation of the catalytic cysteine. Bacterial cells were harvested, and cell lysates were incubated with NTA beads. Ubiquitylated proteins were detected by Western blot. **(C)** Flag-DCAF1/Myc-CUL4 complexes were expressed in HEK293T cells for 48 h and immobilized on α-Flag M2 beads. In vitro ubiquitylation assays were performed with 200 nM MBP-PLK4, 170 nM UBA1, 1 μM UBCH5C, 30 μM ubiquitin, 5 mM ATP, and immobilized Flag-DCAF1 complexes for 90 min at 37°C. For better visualization, the Western blot membrane has been cut before detection and exposed for different times. **(D)** HA-ubiquitin, Flag-PLK4, and Myc-DCAF1 were overexpressed in HEK293T cells for 24 h with or without inhibition of Cullin-RING E3 ligases by treatment with 5 μM MLN4924 for 5 h before harvest. The 26S proteasome was blocked by 10 μM MG132 for 5 h before harvest. Flag-PLK4 was immunoprecipitated from cell lysates in the presence of 10 mM *N*-ethylmaleimide using α-Flag M2 beads. Quantification of relative HA-ubiquitin/Flag-PLK4 signal normalized to the −DCAF1 control, N = 3 independent experiments for −DCAF1 and +DCAF1, and N = 2 experiments for MLN4924. *P < 0.05. Data are presented as the mean ± SD.

To assess whether the DCAF1-PLK4 interaction is indeed direct, we performed an in vitro binding assay using purified PLK4 and DCAF1 proteins. We could clearly show that PLK4 and DCAF1 bind to each other in vitro (Fig 4A). To further prove a direct interaction, we used in vitro ubiquitylation assays in a heterologic environment that also lacks possible mediator components. We used two different in vitro ubiquitylation assays: (1) we reconstituted the DCAF1-dependent PLK4 ubiquitylation cascade in *E. coli* (Keren-Kaplan et al, 2012). To circumvent the complexity and the tight regulations of the mammalian system, in the *E. coli*-based, constructed system, the E2 enzyme was fused to the substrate receptor DCAF1 and co-expressed with GFP-PLK4, E1, and His_6_-ubiquitin. We found that PLK4 underwent ubiquitylation when a full cascade was reconstructed. However, strains that expressed only E1, E2-DCAF1, or only PLK4 or a complete cascade but containing a catalytic mutation in the E2 sequence (C86A) did not yield PLK4 ubiquitylation (Fig 4B). (2) We performed an in vitro ubiquitylation assay with recombinant PLK4 and observed ubiquitylation of PLK4 in the presence of UBA1 (E1), UBCH5C (E2) (Han et al, 2020), and DCAF1 WT but not DCAF1 ΔWD40-ΔAcidic, which lacks the PLK4-binding domain (Fig 4C).

To further corroborate that the regulation of PLK4 by DCAF1 is mediated by ubiquitylation, we also performed in vivo ubiquitylation assays. We found that the overexpression of DCAF1 led to an increase in PLK4 polyubiquitylation (Fig 4D). This effect was reversed by inhibition of Cullin-RING E3 ubiquitin ligases with the small-molecule neddylation inhibitor MLN4924. Together, our data show that PLK4 and DCAF1 form complexes in vivo and in vitro, revealing that PLK4 is a new substrate of the CRL4^DCAF1^ E3 ubiquitin ligase complex.

### DCAF1 binds and ubiquitylates PLK4 predominantly in the G2 phase

**Figure 5.**
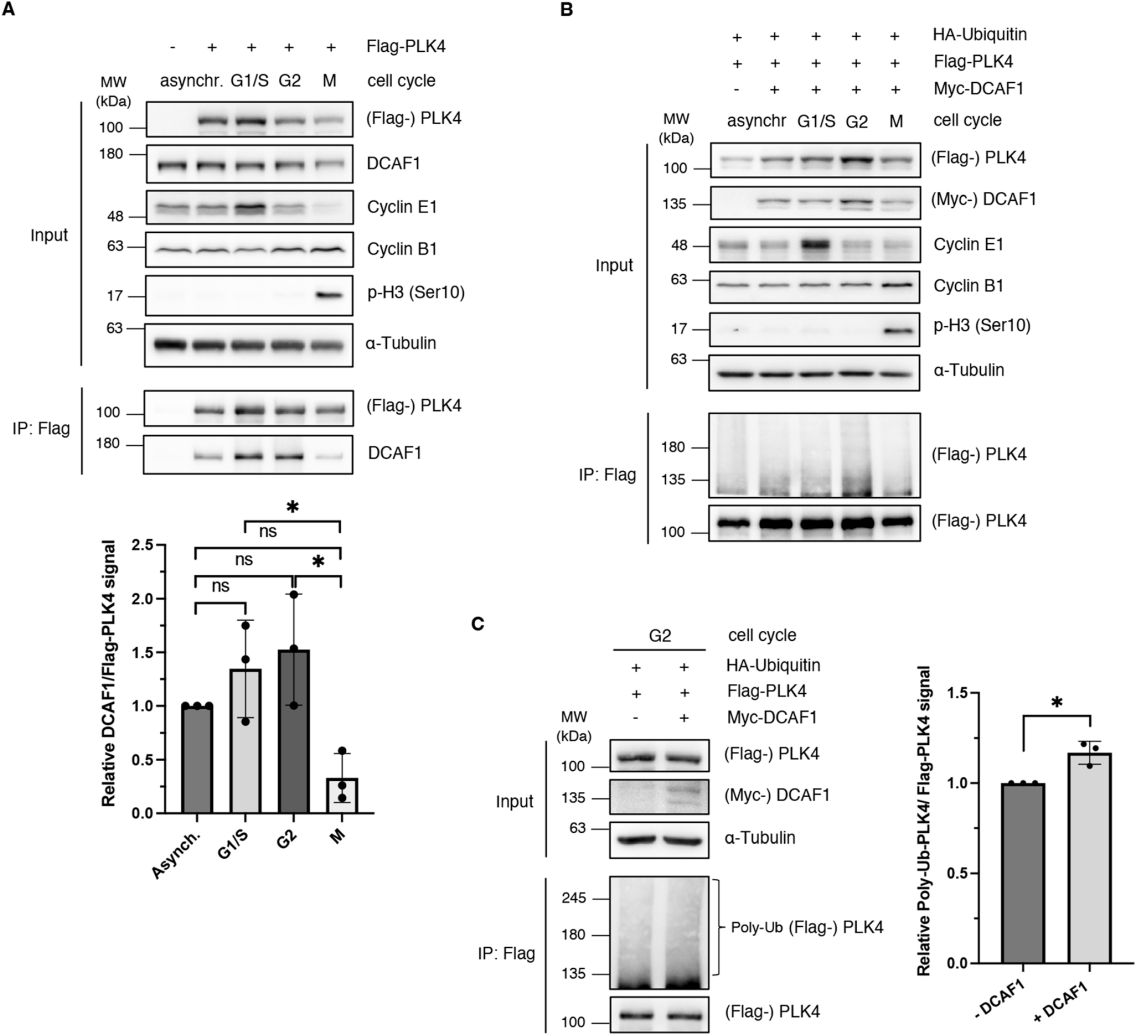
DCAF1 interacts with and ubiquitylates polo-like kinase 4 (PLK4) predominantly in the G2 phase of the cell cycle. **(A)** Flag-PLK4 was overexpressed in HEK293T cells for 48 h. Cells were synchronized in the G1/S phase by double thymidine arrest, in the G2 phase by CDK1 inhibition with RO-3306, or in the M phase by single thymidine and nocodazole arrest, as indicated. Flag-PLK4 was immunoprecipitated from cell lysates using α-Flag M2 beads. Quantification of relative DCAF1/Flag-PLK4 signal normalized to asynchronous cells, N = 3 independent experiments. *P < 0.05 and ns *P* > 0.05. Data are presented as the mean ± SD. **(B)** HA-ubiquitin, Flag-PLK4, and Myc-DCAF1 were overexpressed in HEK293T cells for 24 h. Cells were synchronized as in (A) and treated with 10 μM MG132 for 5 h before harvest. Flag-PLK4 was immunoprecipitated from cell lysates in the presence of 10 mM *N*-ethylmaleimide using α-Flag M2 beads. For better visualization, the Western blot membrane has been cut before detection and exposed for different times. **(C)** HA-ubiquitin, Flag-PLK4, and Myc-DCAF1 were overexpressed in HEK293T cells for 24 h. Cells were synchronized in the G2 phase by CDK1 inhibition with RO-3306. To inhibit the 26S proteasome, cells were treated with 10 μM MG132 for 5 h before harvest and Flag-PLK4 was immunoprecipitated from cell lysates in the presence of 10 mM N-ethylmaleimide using α-Flag M2 beads. Quantification of relative Poly-Ub-Flag-PLK4/Flag-PLK4 signal normalized to the −DCAF1 control, N = 3 independent experiments. *P < 0.05. Data are presented as the mean ± SD.

In mammalian cells, PLK4 is binding to its centriole receptors CEP152 and CEP192, which encircle the proximal end of the parent centriole to initiate centriole duplication at the G1/S phase transition (Cizmecioglu et al, 2010; Hatch et al, 2010; Kim et al, 2013; Sonnen et al, 2013; Park et al, 2014). Activation of PLK4 at the centriole occurs through trans-autophosphorylation (Holland et al, 2010). Phosphodegrons generated in response to PLK4 autophosphorylation are recognized by SCF^β-TrCP^ (Guderian et al, 2010; Cunha-Ferreira et al, 2013), which triggers PLK4 degradation (Klebba et al, 2013). As the regulation of PLK4 by CRL4^DCAF1^ is independent of phosphorylation and DCAF1 binds to the PB1-PB2 domain of PLK4, we asked at what time in the cell cycle PLK4 protein levels are regulated by CRL4^DCAF1^ and whether this timing would be different from the regulation of PLK4 by SCF^β-TrCP^ at the G1/S phase transition. To address this question, we expressed PLK4 in cells that were subsequently synchronized and observed an interaction between DCAF1 and PLK4 during interphase, which was reduced in mitosis (Fig 5A). Next, we analyzed whether the cell cycle–regulated interaction between PLK4 and DCAF1 correlates with ubiquitylation of PLK4 by CRL4^DCAF1^. Using cells that were synchronized in G2 by the CDK1 inhibitor RO-3306, we could show that PLK4 is predominantly ubiquitylated in the G2 phase of the cell cycle (Fig 5B). Quantification of increased ubiquitylation in the G2 phase can be clearly attributed to DCAF1 (Fig 5C). Together, these results suggest that the CRL4^DCAF1^ complex regulates protein levels of PLK4 in the G2 phase, at a time when SCF^β-TrCP^ E3 ligase activity is low, as shown by Paul et al (2022).

### CRL4^DCAF1^ prevents premature binding of PLK4 to STIL

**Figure 6.**
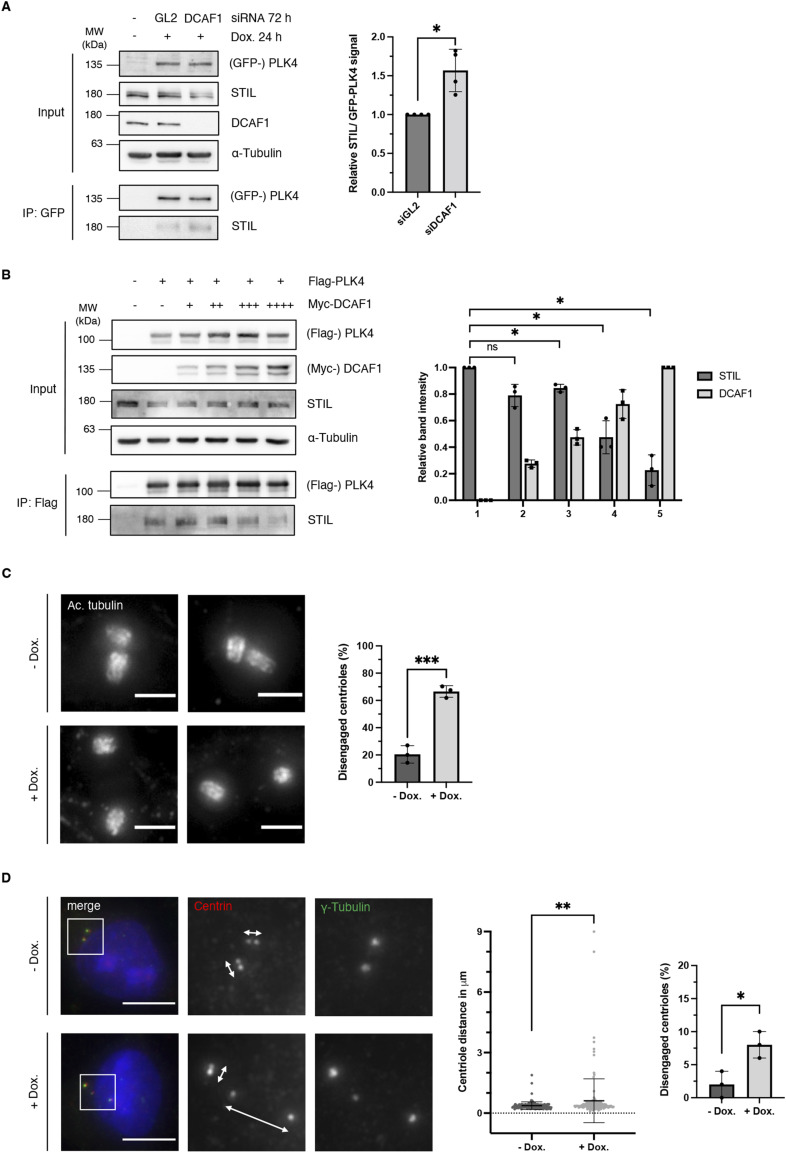
DCAF1 regulates the interaction of polo-like kinase 4 (PLK4) with its substrate STIL and is required to prevent premature centriole disengagement in the G2 phase. **(A)** HeLa tet-on GFP-PLK4 cells were transfected twice every 24 h with 40 nM siRNA targeting GL2 (control) or DCAF1. The overexpression of GFP-PLK4 was induced by treatment with 2 μg/ml doxycycline for 24 h before harvest. GFP-PLK4 was immunoprecipitated from cell lysates using GFP-trap beads. Quantification of relative STIL/GFP-PLK4 signal normalized to GL2 (control), N = 4 independent experiments. *P < 0.05. Data are presented as the mean ± SD. **(B)** Flag-PLK4 was overexpressed in HEK293T cells together with different amounts of Myc-DCAF1 plasmid DNA in different samples (+, ++, +++, ++++) for 48 h. Co-precipitated STIL was detected by IP against the Flag tag and subsequent Western blot analysis. Quantification of relative STIL/Flag-PLK4 and Myc-DCAF1 signal, N = 3 independent experiments. *P < 0.05 and ns *P* > 0.05. Data are presented as the mean ± SD. **(C)** For knockdown of DCAF1, HeLa tet-on shDCAF1 cells were treated with 2 μg/ml doxycycline for 72 h before fixation. G2 arrest was induced by treatment with the CDK1 inhibitor RO-3306 for 18 h before fixation. Representative expansion microscopy images of centrioles stained against acetylated tubulin. Scale bar: 3 μm (physical scale), 0.68 μm (biological scale). Quantification of the percentage of cells with disengaged centrioles in the G2 phase. Distances of more than one centriole length between the two centrioles of a centriole pair were considered as disengaged. N = 3 independent experiments with n = 37, 42, and 40 centriole pairs analyzed per condition. ***P < 0.001. Data are presented as the mean ± SD. **(D)** For knockdown of DCAF1, HeLa tet-on shDCAF1 cells were treated with 2 μg/ml doxycycline for 72 h before fixation. For immunofluorescence analysis, cells were stained with antibodies against γ-tubulin and centrin. White arrows indicate the distance between centrioles. Scale bar: 10 μm. Middle panel: centriole distance values from N = 3 independent experiments with n = 50 centriole pairs analyzed per condition for each experiment. **P < 0.01. Data are presented as the mean ± SD. Right panel: quantification of the percentage of cells with disengaged centrioles. Distances of more than 0.75 μm between the two centrioles of a centriole pair were considered as disengaged. *P < 0.05. Data are presented as the mean ± SD.

**Figure S8.**
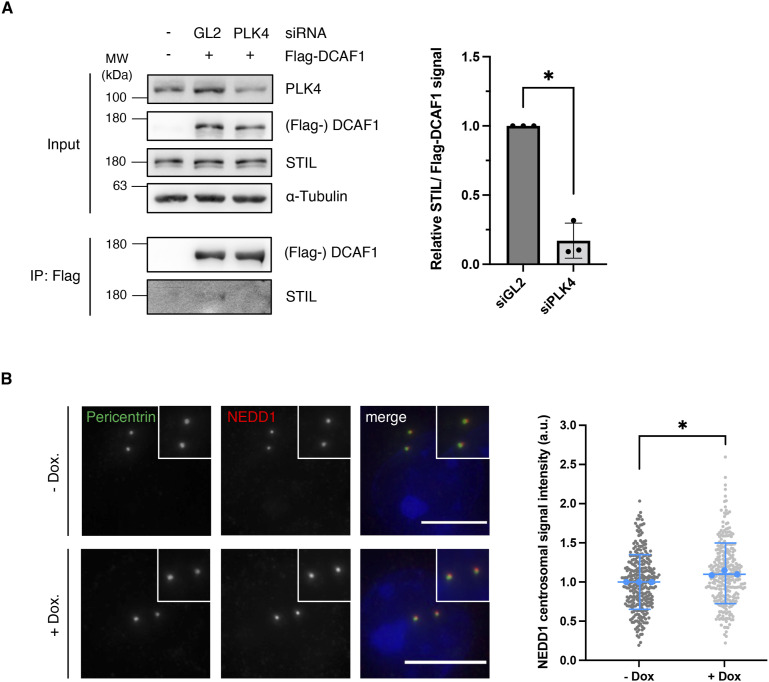
DCAF1 depletion leads to increased levels of NEDD1 at the centrosome. **(A)** HEK293T cells were transfected twice with siRNA against either GL2 (control) or polo-like kinase 4. 48 h after the first siRNA transfection, Flag-DCAF1 was overexpressed for 24 h. Co-precipitated STIL was detected by IP against the Flag tag and subsequent Western blot analysis. Quantification of relative STIL/Flag-DCAF1 signal, N = 3. *P < 0.05. Data are presented as the mean ± SD. **(B)** For knockdown of DCAF1, HeLa tet-on shDCAF1 cells were treated with 2 μg/ml doxycycline for 72 h before fixation. For immunofluorescence analysis, cells were stained with antibodies against pericentrin and NEDD1. Scale bar: 10 μm. Centrosomal signal intensities were quantified, and background fluorescence intensity was subtracted. Values were normalized to the untreated control. Individual values are presented with the mean ± SD. In total, n = 300 centrosomes per condition were analyzed in N = 3 independent experiments. Statistical analysis of the mean values of three experiments. *P < 0.05.

We then aimed at deciphering a possible function of DCAF1 in regulating PLK4 protein levels. Because we found that DCAF1 ubiquitylates PLK4 in the G2 phase, we asked whether it would exert a role in the G2 phase to possibly regulate PLK4 functions in the mitosis/early G1 phase. During mitosis, the mitotic kinase CDK1/cyclin B binds STIL and prevents the formation of the PLK4-STIL complex and STIL phosphorylation by PLK4, thus inhibiting the untimely onset of centriole biogenesis (Zitouni et al, 2016). It is conceivable that DCAF1 may prevent premature binding of PLK4 to STIL in the G2 phase by keeping PLK4 levels low. To find out whether depleting DCAF1 would affect the complex formation of PLK4 and STIL, we analyzed the amount of STIL binding to PLK4 in the presence and absence of DCAF1. We found that upon depletion of DCAF1, a higher amount of STIL binds to PLK4 (Fig 6A). To confirm this finding, instead of depleting DCAF1, an increasing amount of DCAF1 was co-expressed along with PLK4 in cells and the level of STIL binding to PLK4 was assessed. We found that an increase in DCAF1 levels leads to a significant decrease in the amount of STIL binding to PLK4 (Fig 6B). Direct binding of DCAF1 to STIL itself is unlikely as the weak interaction that we observed is most likely mediated by PLK4 and reduced when PLK4 is depleted (Fig S8A). These findings indicate that DCAF1 has a vital regulatory function not only for PLK4 itself but also for the interaction between PLK4 and its substrate STIL further downstream. Moreover, depletion of DCAF1 also affects the levels of the PLK4 substrate NEDD1 at the centrosome (Chi et al, 2021) (Fig S8B).

After exit from mitosis and entry into G1, the centrioles are “licensed” for a subsequent round of centriole duplication as the engaged centriole pairs lose their tight orthogonal configuration leading to centriole disengagement (Tsou & Stearns, 2006). Premature disengagement of centrioles in the absence of DCAF1 should lead to premature centriole reduplication. We used premature centriole disengagement in the G2 phase as a readout for perturbed timing of centriole reduplication. Indeed, we found that doxycycline-induced knockdown of DCAF1 leads to a significantly higher number of disengaged centrioles in cells synchronized in the G2 phase (Fig 6C). To further demonstrate the effect on centriole disengagement in asynchronous cells, independently from interference with the CDK1 function, we measured intercentriolar distances between two centrioles of a centriole pair in HeLa cells with four centrioles. In the absence of DCAF1, we found significantly increased intercentriolar distances and a higher number of cells with disengaged centrioles (Fig 6D), indicating premature centriole disengagement leading to premature centriole reduplication as a cause for the supernumerary centrioles observed upon DCAF1 knockdown. Together, these results suggest that controlled PLK4 protein levels in the G2 phase are necessary to prevent unscheduled centriole duplication by preventing premature interaction between PLK4 and STIL.

## Discussion

**Figure 7.**
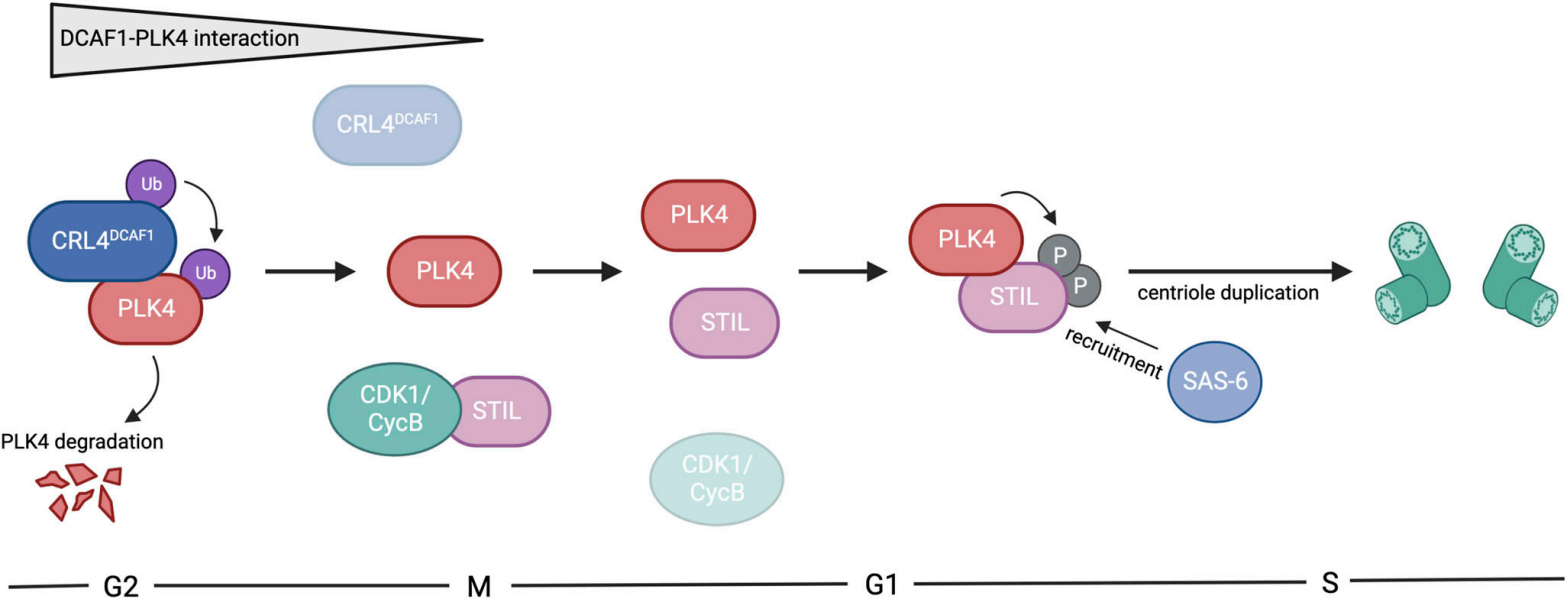
CRL4^DCAF1^ regulates polo-like kinase 4 (PLK4) in centriole duplication. Graphical representation of the proposed involvement of the CRL4^DCAF1^ ubiquitin ligase in centriole duplication. PLK4 interacts with DCAF1 predominantly in the G2 phase of the cell cycle, which leads to ubiquitylation and subsequent proteasomal degradation of PLK4. In mitosis, the strong interaction between PLK4 and DCAF1 is lost, releasing PLK4 to allow for the interaction with STIL. Simultaneously, STIL is bound to CDK1/cyclin B. Upon mitotic exit, PLK4 competes with CDK1/cyclin B for the interaction with STIL. STIL is released from binding to CDK1/cyclin B, now also allowing for the interaction with PLK4. Once both PLK4 is released from DCAF1 and STIL is released from CDK1, the formation of the PLK4-STIL complex can occur in the early G1 phase. This in turn leads to phosphorylation of STIL by PLK4 and the recruitment of SAS-6 for the new round of centriole duplication in the S phase. This figure was created with BioRender.com.

Extensive research has shown that centrosome number control is critical for the maintenance of genomic integrity. In this study, we introduce an additional layer of regulation that allows the timely removal of PLK4, the master regulator of centriole duplication (Nigg & Holland, 2018; Goundiam & Basto, 2021). Our data indicate that at least two pathways exist that regulate PLK4 protein levels and restrict centriole duplication to once per cell cycle, in order to prevent excess centrosome number. These pathways are governed by two distinct ubiquitylating enzymes, one where the SCF^β-TrCP^ E3 ubiquitin ligase directly recognizes phosphorylated S285/T289 on PLK4 (Guderian et al, 2010; Holland et al, 2010; Cunha-Ferreira et al, 2013; Klebba et al, 2013) and one that is independent of PLK4 autophosphorylation and mediated by the CRL4^DCAF1^ E3 ubiquitin ligase complex. Interestingly, the two pathways do differ not only in respect of the phosphorylation status of PLK4 but also in the different phases of the cell cycle where they regulate PLK4 protein levels. Whereas SCF^β-TrCP^ controls PLK4 levels at the G1/S phase transition, we find that CRL4^DCAF1^ targets PLK4 during the G2 phase to control its levels during a different phase of the cell cycle. However, we cannot rule out that DCAF1 might bind other, additional targets at the centrosome apart from PLK4. Recently, it has been shown, using a fluorescent biosensor to quantitatively measure β-TrCP activity, that β-TrCP is highly active during the quiescent G0 state, moderately active in the G1 phase, and the least active during the S and G2 phase (Paul et al, 2022). Our own data reveal that DCAF1 binds PLK4 predominantly in the G1/S and G2 phase (Fig 5A), but this binding leads to a stronger ubiquitylation of PLK4 only in the G2 phase (Fig 5B and C). From procentriole assembly throughout the S phase until late mitosis, the procentrioles remain in a tight, near-orthogonal association with their parental centrioles. This connection is lost in late mitosis/early G1, during centriole disengagement. The centriole–procentriole engagement is thought to prevent the unscheduled procentriole assembly. We propose that PLK4 protein levels have to be controlled especially in the G2 phase by CRL4^DCAF1^-mediated ubiquitylation and degradation to prevent premature centriole disengagement in G2, a process that is critical for licensing the subsequent round of centrosome duplication (Tsou & Stearns, 2006). The untimely onset of centriole duplication is prevented by the mitotic kinase CDK1/cyclin B that binds STIL and prevents the formation of the PLK4-STIL complex and STIL phosphorylation by PLK4 (Zitouni et al, 2016). Our findings imply that CRL4^DCAF1^ binds and ubiquitylates PLK4 in the G2 phase but not in mitosis. As ubiquitylation of PLK4 by CRL4^DCAF1^ causes degradation of PLK4, we propose a mechanism where low PLK4 levels prevent binding and activation of the PLK4 substrate STIL, thus impeding premature initiation of centriole duplication. CDK1/cyclin B may prevent STIL-PLK4 interaction by binding STIL in a kinase-independent fashion through the same region as PLK4 (Zitouni et al, 2016). It is conceivable that in mitosis, when the interaction between CRL4^DCAF1^ and PLK4 is weak, less PLK4 is ubiquitylated and degraded by CRL4^DCAF1^ resulting in higher levels of PLK4. Upon mitotic exit, STIL is released from binding to CDK1/cyclin B, now also allowing for the interaction with PLK4. Once both PLK4 is released from its interaction with and ubiquitylation by DCAF1 and STIL is released from binding to CDK1, the formation of the PLK4-STIL complex can occur (Fig 7). Thus, keeping PLK4 levels low during the G2 phase and early mitosis by CRL4^DCAF1^ ubiquitylation and degradation may prevent premature complex formation between STIL and PLK4. Therefore, the regulation of PLK4 by CRL4^DCAF1^ represents another important pathway to control PLK4 activation and binding to STIL at the onset of centriole duplication.

We found that the interaction between DCAF1 and PLK4 has also interesting structural aspects. PLK4 is a structurally divergent PLK family member characterized by a single polo-box (PB3), which is capable of intermolecular homodimerization and a conserved central region called “cryptic polo-box” (PB 1-2), which is necessary for its functions in centriole duplication. Because we found that DCAF1 binds PB1-2 of PLK4 (Fig S2A and B), we compared our model of the DCAF1/PLK4 complex (Fig 3A and B) with the CEP192/PLK4 and CEP152/PLK4 complexes, because both CEP192 and CEP152 were previously shown to also bind PB1 and PB2 of PLK4 (Kim et al, 2013; Sonnen et al, 2013). Acidic helical regions of CEP152 and CEP192 bind to PB1-2 of PLK4 in opposite directions (Park et al, 2014). Interestingly, we found that PLK4 binds CEP192 and DCAF1 in a very similar manner via a conserved groove within the PLK4 PB1-PB2 domain (Fig 3B). However, although each of CEP152 and CEP192 presents a short acidic helix, DCAF1 has two short acidic helices connected by a flexible linker that allows them both to bind simultaneously. The first region (D1420-E1436) binds with the same orientation as CEP192, whereas the second region (D1458-E1466) interacts with the same orientation as CEP152. It would be interesting to further investigate a potential correlation between DCAF1 and CEP152/CEP192 in the regulation of PLK4 and centriole duplication.

A common structural mechanism for protein–protein interactions is often achieved by the β-sheet assembly, where one interaction partner donates a single β-strand, and the other partner donates a β-sheet that lacks one or a few strands. Such a phenomenon was previously described as the structural basis for the binding of the Merlin FERM domain to DCAF1 (Li et al, 2014). The terminal β-strand of DCAF1 (amino acids DIILSLN in the β-B) forms a β-sheet with βF5 of the FERM domain. Careful examination of the AlphaFold2.0 model of the DCAF1/PLK4 complex suggests that a similar interaction takes place for the same DCAF1 sequence to extend a PLK4 β-sheet of PB1.

Targeted protein degradation represents an emerging therapeutic modality with the potential to tackle disease-causing proteins that have in the past been highly challenging to target with conventional small molecules. Our results pave the way for the development of CRL4^DCAF1^-dependent PROTACs or molecular glue degraders (Levin-Kravets et al, 2021; Bekes et al, 2022) to target PLK4 degradation as a novel modality for cancer therapy.

## Materials and Methods

### Cell lines, cell culture, and cell cycle synchronization

HEK293T (catalog no.: ACC 635; DSMZ, Braunschweig), HeLa (CCL-2; ATCC), HeLa tet-on shDCAF1, and U2OS (HTB-96; ATCC) cells were cultured in DMEM containing 4.5*g*/liter glucose (catalog no.: 41965-039; Gibco) supplemented with 10% FBS (catalog no.: 10270-106; Gibco) and 1% penicillin–streptomycin (catalog no.: P0781; Sigma-Aldrich) at 37°C and 5% CO_2_.

For the generation of a stable cell line for conditional DCAF1 knockdown, HeLa S/A cells (O. Gruss, ZMBH, Heidelberg) were transiently co-transfected with a pBi-9 vector containing the siDCAF1 shRNA sequence and the pCAGGS-flpE vector (O. Gruss, ZMBH, Heidelberg). Antibiotic selection for successful integration of the construct was performed using 2.5 μg/ml puromycin (Sigma-Aldrich) and 10 μM ganciclovir (Alpha Diagnostic Intl. Inc.). DCAF1 knockdown was induced by the addition of 2 μg/ml doxycycline (catalog no.: D9891; Sigma-Aldrich) for 72 h.

Cullin-RING E3 ubiquitin ligases were inhibited by the addition of 5 μM MLN4924 (catalog no.: 85923; Cell Signaling Technology), and the 26S proteasome was blocked with 10 μM MG132 (catalog no.: C2211; Sigma-Aldrich) for 5 h before harvest.

For double thymidine arrest of HEK293T cells in the G1/S phase, cells were treated with 2 mM thymidine (catalog no.: sc-296542A; Santa Cruz Biotechnology) for 18 h, released for 9 h, and arrested again for 16 h. For single thymidine and nocodazole arrest in mitosis, cells were treated with 2 mM thymidine for 20 h, released for 2 h, and treated with 100 ng/ml nocodazole (Merck) for 16–17 h. For G2 phase arrest of HeLa or HEK293T cells, cells were treated with 10 μM RO-3306 for 18 h.

### Plasmids, cloning, and mutagenesis

pCMV-3Tag1A-Flag-PLK4 full-length and fragments have been described previously (Cizmecioglu et al, 2010). pCMV-3Tag1A-Flag-PLK4 mutants were generated by site-directed mutagenesis. pCMV-Sport6-Flag-DCAF1 full-length and fragments were a gift from Prof. Vicente Planelles (Cassiday et al, 2015). pCMV-3Tag2C-Myc-DCAF1 was generated by subcloning using EcoRI and XhoI. For the generation of a stable cell line for inducible knockdown of DCAF1, shRNA constructs were cloned to the pBI-9 vector (O. Gruss, ZMBH, Heidelberg) using BsaI. Constructs for the expression and detection of ubiquitylated PLK4 in *E. coli* were generated by the Gibson assembly (Gibson et al, 2009). EGFP-PLK4_580-808_ was subcloned into pET22b, and the His_6_-tag was removed. DCAF1_1395-1507_ was subcloned into pGEN13 upstream of and in-frame with Ubc4 (Keren-Kaplan et al, 2012). The C86A mutation at the catalytic cysteine of Ubc4 was also generated by the Gibson assembly.

### Plasmid and siRNA transfections

HEK293T cells were transfected with plasmid DNA using polyethylenimine (Polysciences) at a final concentration of 5 μg/ml for 24 or 48 h. HeLa and HEK293T cells were transfected with siRNA using Lipofectamine 2000 (Invitrogen). Cells were transfected with 40 nM of siRNA for 24 and 48 h after seeding and further cultivated for another 48 h after the second siRNA transfection.

The following siRNA sequences were used:

GL2 5′-CGUACGCGGAAUACUUCGADTDT-3′.

DCAF1 #1 5′-UCACAGAGUAUCUUAGAGADTDT-3′ (Nakagawa et al, 2015).

DCAF1 #2 5′-CGGAGUUGGAGGAGGACGAUUDTDT-3′ (Hakata et al, 2014).

DCAF5 5′-GCAGAAACCUCUACAAGAADTDT-3′ (Ambion, silencer select).

DDB1 5′-ACACUUUGGUGCUCUCUUUDTDT-3′ (Ambion).

CUL4A 5′-GACAAUCCGAAUCAGUACCDTDT-3′ (Ambion).

PLK4 5′-GGUAGUACUAGUUCACCUADTDT-3′ (Ambion).

### Cell lysis, co-immunoprecipitation, and Western blot analysis

Cell lysates were prepared as described previously (Hanle-Kreidler et al, 2022). Briefly, cells were harvested and washed with ice-cold PBS. For Western blot analysis, cell pellets were lysed in RIPA buffer (50 mM Tris–HCl, pH 7.4, 1% NP-40, 0.5% sodium deoxycholate, 0.1% SDS, 150 mM NaCl, 2 mM EDTA, and 50 mM NaF), and for immunoprecipitation, cell pellets were lysed in NP-40 buffer (40 mM Tris–HCl, pH 7.5, 150 mM NaCl, 5 mM EDTA, 10 mM β-glycerophosphate, 5 mM NaF, and 0.5% NP-40). Both buffers were supplemented with 1 mM DTT, 10 μg/ml l-1-tosylamido-2-phenylethyl chloromethyl ketone, 5 μg/ml tosyl lysyl chloromethyl ketone, 0.1 mM Na_3_VO_4_, 1 μg/ml aprotinin, 1 μg/ml leupeptin, and 10 μg/ml trypsin inhibitor from soybean. Cell lysates were incubated on ice for 30 min and centrifuged for 20 min at 16,100*g*. For SDS–PAGE, cell extracts were mixed with 2x Laemmli buffer and incubated for 5 min at 95°C. For immunoprecipitation of Flag-tagged proteins, 3–6 mg of cell extract and 20 μl of α-Flag M2 affinity bead suspension (catalog no.: A2220; Sigma-Aldrich) were used. The beads were prepared by washing twice with TBS, once with glycine buffer (0.1 M glycine–HCl, pH 3.5), and thrice with TBS. Cell extracts were incubated with beads for 3 h or overnight on a rotating wheel at 4°C. Before elution, the beads were washed three times with NP-40 buffer. For elution, the beads were incubated with 3x Flag peptide (catalog no.: A36805; Thermo Fisher Scientific) for 30 min on ice with short vortexing for every 5–10 min. Eluates were mixed with Laemmli buffer and denatured for 5 min at 95°C. Proteins were resolved by SDS–PAGE and detected by chemiluminescence after Western blot. For immunoprecipitation of GFP-tagged proteins, cell extracts were incubated with GFP-trap beads. For endogenous immunoprecipitation, cell extracts were incubated with 4 μg of mouse anti-DCAF1 (catalog no.: sc-376850; Santa Cruz Biotechnology) antibody overnight on a rotating wheel. Protein G Sepharose beads were added for 2 h at 4°C. The beads were washed three times with NP-40 buffer, and proteins were eluted by incubation with 2x Laemmli buffer for 5 min at 95°C. Samples were analyzed by SDS–PAGE and Western blot.

### CHX chase assay

For the analysis of PLK4 protein stability in the presence or absence of DCAF1, U2OS cells were transfected twice with siRNA against GL2 (control) or DCAF1 using Lipofectamine 2000. 72 h after the first transfection, 100 μg/ml CHX (ChemCruz, catalog no.: sc-3508; Santa Cruz Biotechnology) was added to block protein synthesis. Samples were harvested at different time points and analyzed by SDS–PAGE and Western blot.

### GST pull-down assay

Recombinant GST-DCAF1 and MBP-PLK4 were expressed in *E. coli* Rosetta (DE3) and purified using glutathione CL-4B Sepharose (Sigma-Aldrich) or amylose resin (NEB). In vitro GST pull-down assays were performed as described previously (Hanle-Kreidler et al, 2022). Briefly, 10 μg of GST-DCAF1 and 10 μg of MBP-PLK4 (Kratz et al, 2015) were incubated in 200 μl NP-40 buffer on a rotating wheel at 4°C for 1 h. 10 μl glutathione Sepharose beads per reaction were resuspended in 200 μl NP-40 buffer, added to the mixture, and incubated on a rotating wheel at 4°C for 2 h. Beads were washed five times with NP-40 buffer, and proteins were eluted by incubation with 25 μl 2x Laemmli buffer at 95°C for 5 min.

### In vivo ubiquitylation assay

HEK293T cells were transfected with the indicated plasmids for 24 h. Cells were treated with 10 μM proteasome inhibitor MG132 for 5 h before harvest. Cullin-RING E3 ligases were inhibited by treatment with 5 μM MLN4924 for 5 h before harvest. Cells were harvested and lysed in NP-40 buffer supplemented with 10 mM *N*-ethylmaleimide (catalog no.: E3876; Sigma-Aldrich). Flag immunoprecipitation was performed as described previously. Whole-cell extracts and eluates were mixed with Laemmli buffer and denatured for 5 min at 95°C. Proteins were resolved by SDS–PAGE and detected by chemiluminescence after Western blot.

### In vitro ubiquitylation assays

For in vitro ubiquitylation assays using a reconstituted ubiquitylation system in *E. coli*, T7 Express Competent *E. coli* were co-transformed with the indicated constructs and grown in 1-liter LB cultures. 0.1 M IPTG was added for induction at OD_600_ = 1.8, and the bacteria were further grown at 18°C for 12 h. Cells were harvested by centrifugation and lysed with lysozyme in the presence of a serine protease inhibitor (AEBSF). After sonication and high-speed centrifugation, the soluble fraction was incubated with Ni-NTA beads and washed three times in batch with 150 mM NaCl and 50 mM Tris–HCl, pH 7.5. SDS loading buffer was added, and the samples were incubated for 10 min at 70°C before SDS–PAGE and Western blot analysis.

Recombinant MBP-PLK4 (Kratz et al, 2015) was expressed in and purified from *E. coli* Rosetta (DE3). UBA1, UBCH5C, and ubiquitin were kind gifts from Frauke Melchior (University of Heidelberg). Flag-DCAF1/Myc-CUL4 complexes were co-expressed in HEK293T cells for 48 h and immunoprecipitated using α-Flag M2 affinity beads as described previously, but proteins were not eluted after washing. Ubiquitylation reactions were performed in 50 mM Tris–HCl, pH 7.5, 100 mM NaCl, 10 mM MgCl_2_, 0.05% NP-40, 1 mM DTT, 0.1 mM Na_3_VO_4_, 1 μg/ml aprotinin, 1 μg/ml leupeptin, and 10 μg/ml trypsin inhibitor from soybean. Flag-DCAF1/Myc-CUL4 complexes immobilized on α-Flag M2 beads were combined with 200 nM MBP-PLK4, 170 nM UBA1, 1 μM UBCH5C, 30 μM ubiquitin, and 5 mM ATP in 20 μl total volume assay buffer. Samples were incubated for 90 min at 37°C and 400 rpm on an Eppendorf Thermomixer comfort 5355. Reactions were terminated by the addition of 2x LDS buffer and incubation for 10 min at 72°C. Samples were analyzed by SDS–PAGE and Western blot.

### Antibodies

Proteins were detected by Western blot using the following antibodies: α-DCAF1 (C-8, catalog no.: sc-376850), α-CUL4 (H-11, catalog no.: sc-377188), α-cyclin E1 (HE12, catalog no.: sc-247), and α-GST (Z-5, catalog no.: sc-459) antibodies were purchased from Santa Cruz Biotechnology; α-tubulin (catalog no.: T5168), α-Flag (catalog no.: F3165), and α-polyhistidine–peroxidase (catalog no.: A7058) antibodies were purchased from Sigma-Aldrich; α-DDB1 (catalog no.: A300-462A), α-CUL4A (catalog no.: A300-739A), α-STIL (catalog no.: A302-442A), and α-CEP192 (catalog no.: A302-324A) antibodies were purchased from Bethyl; α-phospho-H3 (Ser10) (catalog no.: 06-570) antibody was purchased from Merck; α-HA tag (16B12) antibody was purchased from Babco; α-β-TrCP (D13F10, catalog no.: 4394S) antibody was purchased from Cell Signaling Technology; α-CEP152 (P1285, catalog no.: MA5-18285) antibody was purchased from Thermo Fisher Scientific; the α-PLK4 and α-cyclin B1 antibodies have been described previously (Hoffmann et al, 1993; Cizmecioglu et al, 2010); and the α-DCAF5 antibody was generated by Innovagen AB, Lund, Sweden.

For immunofluorescence, α-pericentrin (catalog no.: ab4448) antibody was purchased from Abcam; α-centrin antibody (catalog no.: 04-1624) was purchased from Merck; α-tubulin (catalog no.: T5168), γ-tubulin (GTU-88, catalog no.: T6557), and α-acetylated tubulin (catalog no.: T7451) antibodies were purchased from Sigma-Aldrich; and the α-DCAF1 antibody is mentioned above.

### Immunofluorescence microscopy

Cells grown on coverslips were washed with PBS and fixed with ice-cold methanol for 10 min at −20°C. Cells were washed with PBS again and blocked with 3% BSA and 0.05% Triton X-100 in PBS (PBS-BT) for 30 min at RT. Primary and secondary antibodies were diluted in PBS-BT, and the antibody incubations were performed for 1 h at RT with three washing steps in between. After washing with PBS another three times, coverslips were mounted onto glass microscope slides using ProLong Gold Antifade (Molecular Probes by Life Technologies) with DAPI. Cells were imaged using the Zeiss Observer.Z1 inverted motorized microscope, and images were processed using Fiji software (Schindelin et al, 2012).

### Ultrastructure expansion microscopy (U-ExM)

Expansion microscopy was performed according to the previously published protocol by Gambarotto et al (2019). Cells on coverslips were fixed using ice-cold methanol for 10 min at −20°C. Cells were washed with PBS and incubated in a 0.7% formaldehyde and 1% acrylamide solution for 4 h at 37°C. Gel polymerization on the coverslips was performed using a gelation solution (23% wt/vol sodium acrylate, 10% wt/vol acrylamide, 0.1% wt/vol BIS, 0.5% TEMED, and 0.5% ammonium persulfate in PBS). Gels were allowed to polymerize for 5 min on ice before coverslips were transferred to 37°C for 1 h. After complete polymerization, coverslips with gels were incubated in denaturation buffer (200 mM SDS, 200 mM NaCl, and 50 mM Tris in ddH_2_O) for 15 min at RT to detach the gels from the coverslips. Gels were transferred to 1.5-ml microcentrifuge tubes filled with denaturation buffer and incubated for 90 min at 95°C. Gels were then transferred to beakers filled with ddH_2_O and washed with ddH_2_O an additional two times for 10 min each. Before antibody labeling, gels were shrunk by replacing ddH_2_O with PBS, washed twice with PBS for 15 min each, and then transferred into a six-well plate. Primary antibodies were diluted in 2% BSA in PBS and added to the gels. Primary antibody staining was performed in the six-well plate overnight at 4°C with agitation. Gels in the six-well plate were washed three times for 10 min each with 0.1% Tween-20 in PBS (PBST) at RT with agitation. Secondary antibodies were diluted in 2% BSA in PBS, added to the gels, and incubated for 2.5 h at 37°C with agitation and protection from light. Gels were washed in the six-well plate as before, transferred to beakers filled with about 1,000 ml ddH_2_O, and incubated in the dark until mounting and imaging.

### Live-cell imaging

Cell division of HeLa shDCAF1 cells with or without doxycycline-induced knockdown of DCAF1 was analyzed by live-cell imaging. 48 h after doxycycline induction, cells were seeded to eight-well imaging dishes at 40% confluency and cultivated in the presence of 2 μg/ml doxycycline for another 24 h. For imaging, the dish was placed in a microscopy incubation chamber at 37°C and 5% CO_2_ and cells were monitored using the Zeiss Observer.Z1 inverted motorized microscope and a 10/0.3 EC PlnN Ph1 DICI objective. Phase-contrast images were taken at multiple positions in each well every 10 min for up to 60 h. Images were analyzed using Fiji software (Schindelin et al, 2012).

### Mass spectrometry analysis of Flag-PLK4 interaction partners

For identification of Flag-PLK4–interacting proteins, Flag-PLK4 elution fractions were resolved by SDS–PAGE and co-precipitating proteins were detected in the gel by staining with colloidal Coomassie. Analysis was performed by M. Schnölzer/DKFZ Protein Analysis Facility (Heidelberg) as described (Kratz et al, 2015). In brief, the gel lanes were cut into slices, digested with trypsin after reduction and alkylation of cysteines. Tryptic peptides were analyzed by nanoLC-ESI-MS/MS using a nanoAcquity UPLC system (Waters GmbH) coupled online to an LTQ Orbitrap XL mass spectrometer (Thermo Fisher Scientific). Data were acquired by scan cycles of one FTMS scan with a resolution of 60,000 at m/z 400 and a range from 300 to 2,000 m/z in parallel with six MS/MS scans in the ion trap of the most abundant precursor ions. Instrument control, data acquisition, and peak integration were performed using Xcalibur software 2.1 (Thermo Fisher Scientific). Database searches were performed against the SwissProt database with the taxonomy “human” using the MASCOT search engine (version 2.2.2; Matrix Science). MS/MS files from the individual gel slices of each lane were merged into a single search. Peptide mass tolerance for database searches was set to 5 ppm, and fragment mass tolerance was set to 0.4 Da. The significance threshold was *P* < 0.01. Carbamidomethylation of cysteine was set as a fixed modification. Variable modifications included oxidation of methionine and deamidation of asparagine and glutamine. One missed cleavage site in case of incomplete trypsin hydrolysis was allowed.

### Statistical analysis

All statistical analyses were performed with GraphPad Prism, version 9 (GraphPad Software, Inc.). Data were collected from at least three independent experiments and represented as individual values or as the mean ± SD. For statistical analysis of fold change data, values were normalized to a control group and a logarithmic transformation was performed in order to ensure that the data are normally distributed. Statistical significance of these data was analyzed by a one-sample, two-tailed *t* test against the mean of the control group, which was set to 1.0, or by a paired, two-tailed *t* test for comparisons among the test groups. Statistical significance of normally distributed data, which were not normalized to a control group, was analyzed by an unpaired, two-tailed *t* test with Welch’s correction. *P*-values below 0.05 were considered statistically significant (ns *P* > 0.05, *P < 0.05, **P < 0.01, ***P < 0.001, and ****P < 0.0001).

## Supplementary Material

Reviewer comments
